# Characterization of peri-infarct zone by CMR is a robust predictor of major adverse events and is strongly associated with systemic inflammatory response post-myocardial infarction

**DOI:** 10.1186/1532-429X-13-S1-P180

**Published:** 2011-02-02

**Authors:** Andrei Sposito, Otavio R Coelho-Filho, Joalbo M Andrade, Ana Laura R Araújo, Dulcineia SP Abdalla, Eliana Cotta Faria, Rob J van der Geest, Jose C Quinaglia Silva, Otavio R Coelho, Jose AF Ramires, Michael Jerosch-Herold, Raymond Y Kwong

**Affiliations:** 1 Faculty of Medical Sciences, State University of Campinas (Unicamp), Campinas, Brazil; 2 Brigham and Women's Hospital, Boston, MA, USA; 3 University of Brasilia (UnB), Brasilia, Brazil; 4 Leiden University Medical Center, The Netherlands, Leiden, Netherlands; 5 Heart Institute (InCor), University of Sao Paulo, Sao Paulo, Brazil

## Introduction

While previous studies suggest that the peri-infarct zone (PIZ) may be an important arrhythmogenic substrate and may be associated with an unfavorable outcome post-MI, to date, there is no convincing mechanistic explanation to support that relationship. Systemic inflammatory response (SIR) in the acute phase post-MI has also been associated an adverse prognosis.Furthermore, a strong SIR may lead to greater heterogeneity of the infarcted myocardium. We hypothesized that the PIZ extent would be associated with the severity of the post-MI SIR. We further sought to determine if prognostic information provided by PIZ extent and SIR would complement classical markers of adverse outcome post-MI.

## Method/results

**Figure 1:**
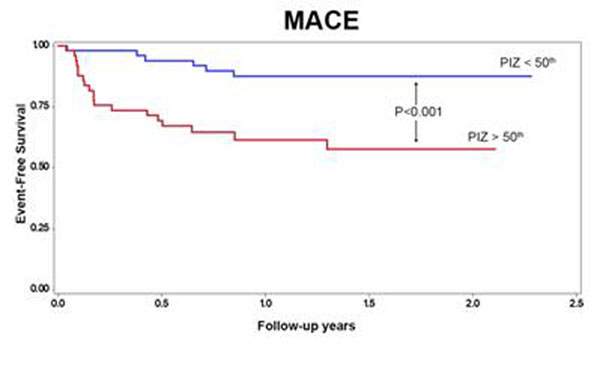


We prospectively enrolled 102 patients (24 females, mean age 55±10) admitted with a ST elevation MI. SIR was estimated on admission (D1) and on the 5^th^ post-MI day (D5) by levels of high-sensitivity CRP, interleukin-2 (IL-2) and tumor necrosis factor type-α (TNF-α). CMR was performed 4-weeks after MI(1.5-TGE-CV/i) including cine imaging, and LGE 10-minutes after a cumulative dose of 0.2mmol/Kg of gadolinium. PIZ, total and core infarct mass were quantified based on signal-intensity (SI) thresholds using the full-width at half-maximum method (tissue with SI>peak remote but<50% of max was defined as PIZ). At a median follow-up of 16.1 months (IQR12.3), 25 major adverse cardiovascular events (MACE)(24%) had occurred (11 cardiac deaths, 8 MIs and 6 unstable anginas). CRP, IL-2 and TNF-α measured at D5 and the variation between D1 and D5 (delta) demonstrated a strong association with PIZ (CRP-D5, r=0.69, p<0.0001; delta-CRP, r=0.7, p<0.0001; IL-2-D5, r=0.5, p<0.0001; delta-IL-2, r=0.6, p<0.0001; TNF-α, r=0.5, p<0.0001; delta-TNF-α, r=0.4, p=0.0001). After adjustment for age, total infarct size, gender and for their respective baseline level, delta-CRP, delta-IL-2 and delta-TNF-α maintain strong association with the PIZ mass expressed as tertiles (delta-CRP, R^2^=0.5, p<0.0001; delta-IL-2, R^2^=0.3, p=0.02; delta-TNF-α, R^2^=0.5, p=0.003). By univariable analysis, the PIZ mass, total infarct size, delta-CRP and delta-IL2 showed the strongest association with MACE(PIZ, LRχ^2^ 25.7, HR 1.13, P<0.0001; total infarct mass, LRχ^2^ 7.2, HR 1.03, P=0.006; delta-CRP, LRχ^2^ 25.3, HR 1.15, P<0.0001; delta-IL2, LRχ^2^ 19.3, HR 1.08, P<0.0001). After adjustment for age, LVEF and delta-CRP, PIZ mass maintained a strong adjusted association with MACE(adjusted LRχ^2^ 8.5, HR 1.17, P=0.003). Patients with PIZ mass >50^th^ demonstrated a significantly reduced MACE-free survival (p=0.0008, Figure 1)

## Conclusion

PIZ as evidence of infarct tissue heterogeneity provides prognostic information post-MI. In addition, the extent of PIZ by CMR is strongly associated with SIR in the subacute phase post-MI.

